# An Energy-Efficient and Adaptive Channel Coding Approach for Narrowband Internet of Things (NB-IoT) Systems

**DOI:** 10.3390/s20123465

**Published:** 2020-06-19

**Authors:** Emmanuel Migabo, Karim Djouani, Anish Kurien

**Affiliations:** 1 Department of Electrical Engineering, Tshwane University of Technology, Pretoria 0001, South Africa; DjouaniK@tut.ac.za (K.D.); KurienAM@tut.ac.za (A.K.); 2 Laboratoire Images, Signaux et Systemes, University of Paris Est Creteil (UPEC), 94400 Creteil, CEDEX, France

**Keywords:** link adaptation, adaptive, energy efficiency, data rates, throughput, Modulation Coding Scheme (MCS), repetition number, Narrowband IoT (NB-IoT)

## Abstract

Most of the current research work on the Narrowband Internet of Things (NB-IoT) is focused on enhancing its network coverage. Many of the existing NB-IoT channel coding techniques are based on repeating transmission data and control signals as a way of enhancing the network’s reliability and therefore, enabling long-distance transmissions. Although most of these efforts are made at the expense of reducing the energy consumption of the NB-IoT network, they do not always consider the channel conditions. Therefore, this work proposes a novel NB-IoT Energy-Efficient Adaptive Channel Coding (EEACC) scheme. The EEACC approach is a two-dimensional (2D) approach which not only selects an appropriate channel coding scheme based on the estimated channel conditions (dynamically classified as bad, medium or good from initial based on a periodically assessed BLER performance outcome) but also minimizes the transmission repetition number under a pre-assessed probability of successful transmission (based on the ratio of previous successful transmissions over the total number of transmissions). This results in creating a single mixed gradient based on which a higher or lower Modulating Coding Scheme (MCS) is selected on each transmission. It is aimed at enhancing the overall energy efficiency of the network by dynamically selecting the appropriate Modulation Coding Scheme (MCS) number and efficiently minimizing the transmission repetition number. Link-level simulations are performed under different channel conditions (good, medium, or bad) considerations to assess the performance of the proposed up-link adaptation technique for NB-IoT. The obtained results demonstrate that the proposed technique outperforms the existing Narrowband Link Adaptation (NBLA) as well as the traditional repetition schemes in terms of the achieved energy efficiency as well as network reliability, latency, and scalability.

## 1. Introduction

Low Power Area Networks (LPWANs) are a promising technology of the Internet of Things (IoT) for future wireless communications. In the recent decade, LPWANs have rapidly developed and have been catching significant attention from many researchers around the globe. The authors in [1] identified the Narrowband Internet of Things (NB-IoT) as well as the Long Range (LoRa) as the two leading LPWAN technologies towards enabling the future of the Internet of Things within the licensed and the unlicensed bands respectively. However, more and more network performance issues are observed as the number of connected IoT devices grows rapidly in the past few years. One of such issues is the energy consumption of the network which is directly related to the network’s lifetime as identified in [2].

Narrowband Internet of Things (NB-IoT) is a new narrowband radio technology introduced in the Third Generation Partnership Project release 13 for providing low-power wide-area IoT [3]. Several techniques that consider the performance enhancement of NB-IoT wireless communication systems are being studied in current research. Specifically, the authors in [4] presented a systematic review of IoT which includes different definitions, key technologies, open issues and major challenges. Furthermore, the authors in [5] provided a systematic survey regarding NB-IoT in industry. Extensive research, key enabling technologies, major NB-IoT applications of IoT in industry, and dentified research trends and challenges were reviewed. At a plenary meeting in South Korea, the Third Generation Partnership Project (3GPP) completed the standardization of NB-IoT in which NB-IoT was regarded as an important technology and a large step for 5G IoT evolution. Major telecommunication companies, including Ericsson, Nokia, and Huawei, have shown great interest in NB-IoT as part of 5G systems and have focused significant effort towards standardization.

In 3GPP standardization, repeating transmission data and the associated control signaling several times has been utilized as a base solution to achieve coverage enhancement for NB-IoT [6]. However, repeated transmissions come with a multiplicative energy cost as it has been demonstrated by several wireless networks energy consumption modeling studies such as [7,8] which showed that the major contributors to the overall average energy consumption of a wireless network are the energy consumed during transmission as well as the energy consumed during reception. This can be represented as

(1) $$E_{Trans}=\frac{(P_{Tx}+P_{Rx})\times M}{B\times P_{st}}$$

where

- $E_{trans}$ is the energy consumed by the transceiver circuit on single wireless link,
- $P_{Tx}$ is the transmission power,
- $P_{Rx}$ is the reception power,
- *M* is the uplink packet size in bits,
- *B* is the transmission bit rate, and
- $P_{st}$ is the probability of successful transmission.

Other previous research work such as [9] have highlighted that the use of NB-IoT link adaptation technique with adaptive modulation and coding schemes as well as adaptive power, in the context of the Long Term Evolution (LTE) would be beneficial to reduce the overall power consumption of the network. This approach however, is limited to the context of using the QPSK modulation scheme to ensure low complexity. Other approaches such as [10,11] propose the use of a scheduler for the NB-IoT system as a mechanism to enhance its overall average delay, to ensure efficient energy and other resources usage and to reduce nodes’ processing time. Finally, authors in [12] propose the repeated transmission approach coupled with efficient bandwidth allocation as a mechanism to achieve energy efficiency.

This article seeks to mitigate the high energy consumption by proposing an adaptive energy-efficient channel coding approach. It is also important to note that the choice of different Modulation Coding Scheme (MCS) levels considerably influences the overall network performance of the NB-IoT system in terms of its reliability, its energy efficiency, its scalability, and latency. This is because the higher the modulation coding scheme, the higher the transmission power per modulated bit of information (lower energy efficiency) but the higher the resilience of the transmitted packet to noise and other channel impairments (more transmission reliability). The use of low MCS coupled with high transmitting power has been demonstrated to improve the transmission reliability and therefore, enhance the network coverage in terms of longer transmission distance and immunity to noise [13]. However, this results in the reduction of the network’s throughput, and causes the overall energy consumption of the network to be significantly high. Although the number of repeated re-transmissions is significantly cut-down, the transceiver’s energy consumption as modeled by Equation (2) remains quite high. This model is based on the fact that the NB-IoT receiving node has three operational states. It is either in synchronization mode or in active mode (handling of the received packets) or otherwise in Idle mode. Most of the energy consumption normally occurs in its active state. Medium energy consumption then occurs in its synchronization state, and the lower amount is consumed during its Idle state [14]. The transceiver’s energy consumption during reception $E_{Rx}$ can be represented as

(2) $$E_{Rx}=KP_{Rx}t_{synch}+\sum_{k=1}^{K}P_{sleep}t_{sleep}^{k}+P_{Idle}t_{active}^{K}$$

where

- $t_{synch}$ is the synchronization time,
- *K* is the number of cycles or iterations involved in the synchronization process for connection to be effectively established,
- $t_{sleep}^{k}$ is the time spent in the sleep state in a reception cycle *k*,
- $t_{active}^{K}$ is the total active time during all the *K* cycles, and
- $P_{Rx},\;P_{sleep}$ and $P_{Idle}$ are the power value for the receiving, sleeping and idle states, respectively.

While the repeating of transmission data or control signals has been selected as a promising approach to enhance coverage of NB-IoT systems according to the 3GPP rel 13 because a larger repetition number would enhance the transmission reliability, it however results in a significant spectral efficiency loss [15].

To overcome this, this article proposes a 2-dimensional channel coding and link adaptation scheme capable of providing a trade-off between the transmit reliability and the throughput of the system by selecting a suitable MCS on one hand and an appropriate transmission repetition number on the other hand. This is proposed based on the fact that it has been identified that most existing link adaptation schemes found in the literature are solely focused on the selection of a suitable MCS value without consideration of the repetition number which as demonstrated in the paragraphs above is has demonstrated potential benefits towards addressing the energy efficiency of the NB-IoT network.

## 2. Methods/Experimental Approach

In this article, key technologies in uplink scheduling and the design of an uplink link adaptation scheme for NB-IoT systems are focused on. The remainder of the article is organized as follows: First, in Section 3, a comprehensive literature survey that concisely and critically discusses the different existing channel coding and MCS selection approaches found in literature is provided. Section 4, then presents and discusses the proposed algorithm as well as the modelling. This section is then followed by a detailed description of the methods used for validation of the proposed approach in Section 5. In this section, a comprehensive table of key simulation parameters is provided, and the different design considerations and assumptions are presented and justified. A step-by-step description of the research design using a simulation script written in the R2019b version of MATLAB and its LTE toolbox, is presented in Section 5.2 before the obtained results are presented in Section 5.3 and critically analyzed and discussed. In this section various simulation results using MATLAB are presented to demonstrate the effectiveness of the proposed scheme. The existing approaches are compared under the same setting to show the strength of the proposed EEACC scheme compared to the traditional repetition-based as well as the NBLA schemes. Finally, conclusions are drawn in Section 6 based on the research objectives and a recommendation for future work is formulated.

### 2.1. Power Consumption Model

The stochastic energy consumption model analytically developed for the NB-IoT network at an NB-IoT node can be represented as follows. Traditionally, energy consumption in NB-IoT is often analyzed in terms of its average value [16]. The energy in an NB-IoT is consumed at different levels and through different processes describing the network operation. For every communication in an NB-IoT, part of the energy is consumed by the electronic circuitry of the transmitting node while another important part is consumed in the transmission process at the sender NB-IoT node in the case of an upload operation. Finally, energy is consumed in the reception process at the receiving node in the case of a download operation.

#### 2.1.1. The Processing Energy Consumption

During the execution of its task, the processor operates in general either in the active, sleep or idle state, and dissipates different amounts of energy in each of them. Its energy dissipation can be modeled at two levels namely:

(a) During a specific operational state: data processing state (active), idle mode and even sleep mode.
(b) During state transition time.

Determining the energy consumption at the processor’s level is not a deterministic experiment but a stochastic one. The process depends on multiple aspects especially the randomness of the duration quantity which itself is due to the random nature of the type of physical phenomena and events for which the NB-IoT is deployed. A typical example is a scenario in which the NB-IoT nodes are deployed within a water distribution system for leakage detection as in [17]. In such a scenario, there is no certainty on when and where exactly a water leakage occurs.

Part of the major sources of uncertainty of the energy consumption of IoT networks includes the uncertainty of time duration in each of its three power modes and the uncertainty of the number of transitions between the different power modes. During its operation, the Central Processing Unit (CPU) of the NB-IoT node goes through different power mode states. The total energy consumption ($E_{DS}$) of the NB-IoT node’s CPU during *n* active, *m* sleep and *l* idle states can be modeled as

(3) $$E_{DS}=\sum_{k=1}^{n}P_{A}T_{A}\left(k\right)+\sum_{k=1}^{m}P_{S}T_{S}\left(k\right)+\sum_{k=1}^{l}P_{I}T_{I}\left(k\right)$$

where $P_{A},P_{S}$ and $P_{I}$ are the powers consumed by the CPU in each of the three modes (Active, Sleep and Idle) respectively, and $T_{A}\left(k\right),T_{S}\left(k\right)$ and $T_{I}\left(k\right)$ are the randomly varying times of the CPU for each of the different power modes. The power values are computed from the current consumption of each mode and the nominal operating battery voltage.

In general, the processor’s operation in a NB-IoT node goes through five possible state transitions which include the transitions from *Active to Idle*, *Idle to Active*, *Active to Sleep*, *Sleep to Active* and *Idle to Sleep*. In addition to the total energy consumption $E_{DS}$ during the operation of the CPU in the different states, the energy consumed during the above mentioned five possible transition states, referred to as $E_{ST}$, can be modeled as

(4) $$E_{ST}=\sum_{k=1}^{n}F_{TR}\left(k\right)T_{TR}\left(k\right)\frac{\left(P_{IS}\left(k\right)+P_{FS}\left(k\right)\right)}{2}\;$$

where $F_{TR}\left(k\right)$ is the frequency of occurrence of a power state transition as modelled for the different operations (initialization, transmission, information relay, reception, etc.) of the NB-IoT node, $T_{TR}\left(k\right)$ is the duration of a state transition *k*, and $P_{IS}\left(k\right)$ and $P_{FS}\left(k\right)$ are the power consumption in the initial and final states respectively. Equation (4) describes the total energy consumed by a NB-IoT node CPU during its multiple transitions (n transitions) from one of its five operating states (e.g., Active state) to another state (e.g., Sleep state). The energy consumed during state transitions is modelled as the sum of all individual energy consumption if all *n* state transitions as undergone by the node during its operation period. Each of these *n* energy consumption is modelled to be directly proportional to the frequency of occurrence of that particular state transition within the operation duration of the node ($F_{TR}\left(k\right)$), also directly proportional to the duration of the particular state transition under consideration ($T_{TR}\left(k\right)$). The product of $F_{TR}\left(k\right)$ and $T_{TR}\left(k\right)$ brings in the time aspect of the electrical energy formula. As it is known, electrical energy is the product of power and duration, the second part of the formula is the average power consumed by the state transition and is calculated as the numerical average of power consumption during the initial state ($P_{IS}\left(k\right)$) and the final state to which we transit ($P_{FS}\left(k\right)$).

The resultant power used in this case is the average (expected) power of the random change of state process. Equations (3) and (4) are used to compute the CPU’s energy ($E_{PR}$) consumption which may be expressed as

(5) $$E_{PR}=E_{DS}+E_{ST}\;$$

There are existing statistical approaches for quantitatively estimating the relationship between the state transitions of a processor and the corresponding power consumption. Some of the well-known approaches include the Performance Monitoring Counters (hardware Monitoring Counters) and the complementary approach. The latter has been proposed by [18] and simply employs the CPU’s utilization data to perform the estimation. The complementary approach, in addition to the instantaneous power consumption, estimates the statistics Cumulative Density Functions (CDF) and Probability Density Functions (PDF) of the power consumed by a processor and relates it to the statistics of its state transitions.

#### 2.1.2. A Stochastic Energy Consumption Model of the Processor Unit Using a Markov Model

**Figure 1:**
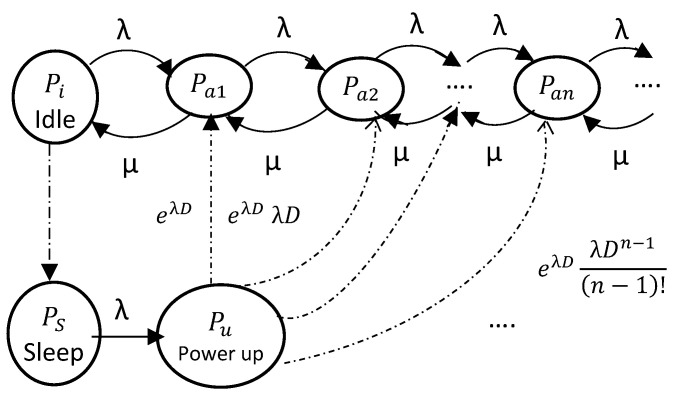
Birth and death process of processor’s tasks.

Consider a processor operation process as illustrated in Figure 1. The processor operates within three states in which it consumes different levels of power. As it operates in sleep mode, it consumes the least amount of energy. When it operates in idle mode, it consumes a medium level of energy. It consumes most of the energy when operated in active mode. Although the aim is to spend the least of time in the Active mode, the amount of time spent in each mode is unpredictable. The proposed Markov model depicts the various increasing states through which the processor passes during its most energy-consuming state (active). Let these states be named pa1, pa2, pa3, etc.

Given a job arrival rate λ at which the different tasks are given to the processor, it services these tasks at a rate μ while strives to move back to the lowest power mode (Sleep mode) or eventually to the idle mode. Statistically, it is proven that most NB-IoT nodes’ processors are designed to spend almost 98% of their time in the sleep mode while they spend roughly 1.5% in the idle state and only 0.5% of their operational time in active mode. Therefore, although all the three operational modes’ times are purely stochastic, the proposed model focuses on modeling the time spent in the active mode, it however also assesses the probability of operational failure which therefore guides the entry into the idle state.

In the proposed model, it is considered that the processor remains in the idle state for some time interval greater than some threshold. The processor then moves back to the sleep mode. In our proposed model, the following assumptions are made

- The request arrivals follow a Poisson process with a mean rate of λ.
- The service time is exponentially distributed with a mean of 1/λ.
- The CPU enters sleep mode if there are no more tasks to be executed for a time interval longer than an energy-aware defined threshold *T* that the network designer heuristically defines depending on the energy consumption objective of the design under consideration versus the network reliability objective.
- The power-up processor of the processor takes a constant time *D* which depends on the type of processor being used.

The operation process of the processor cannot naturally be modelled using the Markov model. Therefore, the method of supplementary variables as proposed in [19] is used to derive an alternative set of state equations to approximate the transitions for stationary analysis. The different mode transitions as highlighted in Figure 1 follow either of the two probabilistic distributions. As the processor transitions from the sleep state to the active mode, the processor goes through a power-up fixed duration *D* which then makes the probability for the processor to stay in each of the active states $P_{a0}$ to $P_{an}$ to exponentially decay by $e^{-\lambda D}$. It is also directly proportional to the parameter $\frac{\lambda D^{n-1}}{(n-1)!}$ for each active state *n*. This implies that the more active states that the processor’s operational process is involved in, the longer the duration it is in the active mode. On the other hand, the duration within the idle mode is any value from a few processor’s machine cycles to a maximum threshold duration of *T*. The duration of the transition from the active mode to the idle mode is directly proportional to the rate of arrival of tasks λ but inversely proportional to the rate at which tasks get services μ. This transition duration can be modeled as

(6) $$T_{ai}=\frac{\lambda \times e^{\lambda T}}{\mu }$$

Considering that the NB-IoT nodes do not stay in any of the states permanently but rather move from one mode to the other, the sum of probability of staying in either mode is equal to unity (certainty). This important constraint is modelled using the following equation

(7) $$\sum_{k=1}^{n}P_{ak}+P_{i}+P_{s}+P_{u}=1$$

#### 2.1.3. The Transceiver Energy Consumption Model

Several research works have confirmed that radio communication dominates the power consumption in NB-IoT above all other functions [20,21,22]. The energy consumption model of the transceiver module follows the same analogy as one of the processors modeled in the previous sub-section. Like the processor, the transceiver also has different operation states. These operational states include the transmission, the reception, the idle, and the sleep states in general. The latter consume different levels of energy. The operation of the transceiver consequently also involves state transitions. By taking into account both the different operational states as well as the states transitions, the total energy consumption of a transceiver can be modeled as

(8) $$E_{Transceiver}=\left(E_{TX}+E_{RX}+E_{Idle}+E_{Sleep}\right)+E_{Trans},$$

where $E_{TX}$, $E_{RX}$, $E_{Idle}$ and $E_{Sleep}$ represent the energy consumed during the transmission, the reception, the idle as well as the sleep states respectively and $E_{Trans}$ is the total energy consumed during the transitions between the states.

In most cases, there are nine possible state transitions from the four power mode states. These include a transition from the transmission to the idle state, and vice versa, the reception to the idle state and vice versa the transmission state to the sleep mode and vice versa; the reception state to the sleep mode and vice versa and the single transition from idle to sleep mode. Taking into account all the nine-state transitions mentioned above the energy consumption $E_{Trans}$ for the probabilistic *n* possible transitions that can occur during a network operation are modeled as

(9) $$E_{Transitions}=\sum_{i=1}^{n}N_{st}\left(j\right)E_{st}\left(j\right)\;$$

where $N_{st}\left(j\right)$ is the frequency of a state transition *j* to occur. The value of this frequency varies with the type of operation that the NB-IoT node is carrying out (relaying information, transmitting or receiving) and $E_{st}\left(j\right)$ is the energy consumed by every state transition *j*. It is also very important to point out that the number of possible transitions between power modes is stochastic and can be modeled as given below. Given that

- The state in which the processor of NB-IoT nodes normally remains in is mostly the sleep mode. This is the state in which it consumes the least of energy possible with most of its internal modules and clocks disabled. However, on event-driven instants, either time events or sensing events, the processor of the NB-IoT nodes will normally wake up and go from sleep mode to active mode to perform transmission or a reception handling activity.
- Upon completion of the activity, it might go to another activity. If all activities are completed successfully, then it would normally go back directly to sleep mode or would momentarily go to the idle mode for a threshold amount of time and only later to sleep mode. This would mean that there would be 3 transitions on successful executions of a single activity and 4 transitions on failed execution of an activity (task) by the processor.
- The number of possible transitions *n* between modes is therefore uncertain and can be modeled as follows
- Given a set of *k* activities to be carried out by the NB-IoT processor and given $P_{s}$ the probability of successful execution of each activity, then the probability of failure of execution of an activity is $1-P_{s}$, and thus
  (10) $$n=3\times k\times P_{s}+4\times k\times \left(1-P_{s}\right)=3\times k\times P_{s}+4\times k-4\times k\times P_{s}=k\times \left(4-P_{s}\right)$$

Based on the above analysis, Equation (8) can be re-written in terms of power consumption as

(11) $$E_{Transceiver}=\left(P_{TX}+P_{RX}\right)\times T_{d}+E_{Idle}+E_{Sleep})+E_{Trans},$$

where $P_{TX}$ and $P_{RX}$ represent the transmit and the receive powers respectively, and $T_{d}$ is the transmission time duration on a single hop which is a probabilistic measure.

The transmission time $T_{d}$ is a random variable which models the time duration for successful transmission on a single link. $T_{d}$ is random due to the unpredictable nature of the wireless channel. However $T_{d}$ remains a function of a few calculated parameters. Some of the main parameters include data size, data rate, and packet collision.

- *Data size (M)*: represents the size of the data being transmitted over the link. This size is variable depending on the type of message being transmitted which could be the interest message, data message, or a control message such as acknowledgment messages (ACK). This quantity is usually measured in bits.
- *Data rate (B)*: This quantity represents the bit rate and is usually measured in bits per second.

Considering an ideal channel condition the transmission time duration can be modelled as

(12) $$T_{d}=\frac{M}{B}\;$$

However, in a practical situation, the time duration could vary from its ideal value as modeled in Equation (12). One of the main random parameters is ***packet collision*** which can be described as follows: In a MAC protocol that makes use of a carrier sense multiple access, after a successful carrier sense has taken place, a collision could occur if another node transmits during the vulnerable period (τ). In a network with a total generated traffic λ, the probability of collision can, therefore, be modeled in terms of Poisson’s distribution with λ being the rate of successful transmission. It can be represented as

(13) $$P_{Coll}=1-e^{-\lambda \tau }.$$

At the network layer, the causes of the difference between the ideal transmission time (minimum transmission time) and the random transmission time duration can be looked at as being caused by re-transmission attempts. In a very broad sense and with some level of abstraction, a random parameter defined as $P_{st}$ (probability of successful transmission) can be defined as

(14) $$T_{d}=\frac{M}{B\times (P_{st})}\;$$

The probability of successful transmission is inversely proportional to the transmission time duration because the higher ($P_{st}\approx 1$), the closer it is to the ideal transmission time duration. In the proposed approach this probability value is statistically modeled as given below.

Due to the stochastic nature of the wireless propagation channel, the probability of successful transmission is uncertain and therefore can be considered to be stochastic. However, in the proposed algorithm, it is computed dynamically and statistically based on the previous transmission success rate. The assumption is that all the uplink transmissions are followed by a downlink packet (either an ACK or NACK downlink packets for the successful and not successful transmission of the packet). The cumulative accounting of ACK and NACK packets on previous transmissions is used to categorize the state of the channel as either bad, medium or good conditions as well as to define the probability of successful transmission $P_{st}$ which is given as follows

(15) $$P_{st}\left(m\right)=\frac{\sum_{1}^{m-1}\left(ACKs\right)}{\sum_{1}^{m-1}\left(ACKs+NACKs\right)}$$

The energy consumption on a single link can, therefore, be modelled as

(16) $$E_{Transceiver}=\frac{(P_{TX}+P_{RX})\times M}{B\times P_{st}}.$$

The energy consumption in the sleep mode ($E_{Sleep}$) and the energy consumption in the idle mode ($E_{Idle}$) can also be modelled in terms of power consumed in each of the two modes multiplied by the duration that the transceiver spends in each of them respectively. The duration of the NB-IoT node in sleep mode is a random variable that can be considered to be exponentially distributed with a mean value of β. The energy consumption during the sleep period can be modelled as

(17) $$E_{Sleep}=P_{Sleep}Expected\left[T_{sleep}\right]$$

where $P_{Sleep}$ is the transceiver’s power consumption during sleep mode which can be computed from the current consumption obtained from the transceiver’s manual, and the nominal battery voltage and $Expected[T_{sleep}]$ is the expected (mean) sleep time duration which can be modelled as

(18) $$Expected\left[T_{sleep}\right]=\int_{-\infty }^{+\infty }T_{sleep}\;pdf\left(T_{sleep}\right)d\left(T_{sleep}\right)\;$$

where $pdf(T_{sleep})$ is the probability density function of the sleep time which can be modelled as

(19) $$pdf\left(T_{sleep},\beta \right)=\beta \;e^{-(\beta \times T_{sleep})}u\left(T_{sleep}\right)\;$$

It is also important to highlight that a significant quantity of energy is consumed in the idle state of the NB-IoT node’s radio operation. This energy cost for a node in idle mode is modeled as approximately equivalent to the energy cost in the receive mode. Therefore, protocols that assume that the receiving and idle power are of little consequence are not efficient for NB-IoT networks. The energy cost during idle listening due to the time spent listening while waiting to receive packets is quite significant. During the idle state, the transceiver can be considered to be just listening to the channel noise (no actual information being received or transmitted), and therefore, consumes $P_{Idle}$ amount of power for a normally distributed random time when there is no significant activity of the radio $T_{Idle}$. The energy consumption of the transceiver during idle state can then be modeled as

(20) $$E_{Idle}=P_{Idle}\times T_{Idle}.$$

#### 2.1.4. Electronic Circuitry Energy Consumption

Though it is negligible in many cases, energy is also consumed by the NB-IoT node’s electronic circuitry. In general, the NB-IoT circuit is made of sensors and signal converters such as ADCs. Most NB-IoT nodes’ circuitry operates either in ***burst*** or ***periodic*** mode. For periodic sensing mode, the NB-IoT node’s energy consumption is almost equal to the sensing energy consumption (meaning when the sensing circuit is “ON”) added with the energy consumed during the “ON” to “OFF” as well as the “OFF” to “ON” state transitions. The electronic circuitry energy consumption can, therefore, be expressed as

(21) $$E_{Circuit}=E_{on-off}+E_{off-on}+E_{Sensing}.$$

## 3. Related Work

Low-Power Wide-Area Network (LPWAN) technologies both in the licensed and in the unlicensed bands such as the NB-IoT, Long Range (LoRa), and many others are striving to become energy efficient over very long distances [23]. NB-IoT is designed for long-life devices and targets a battery life of more than 10 years. To this end, the careful design of smart channel coding schemes has been identified as a potential approach towards enhancing energy-efficiency in NB-IoT [24]. Channel coding is one of the most important aspects of digital communication systems, which can be considered as the main difference between analog and digital systems making error detection and correction possible [25].

In its current form, NB-IoT reuses the LTE design extensively including the numerologies, downlink orthogonal frequency-division multiple-access (OFDMA), uplink single-carrier frequency division multiple-access (SC-FDMA), channel coding, rate matching, interleaving, etc. One of the factors for this extensive reuse of the LTE channel coding is to significantly reduce the time required to develop full NB-IoT specifications [26]. However, some issues are very specific to the NB-IoT network designs among which is the limited energy capacity issue. Therefore, researchers [27,28] have identified a crucial need to develop novel channel coding techniques that are specific to the NB-IoT with different design goals. In this research work, the energy efficiency issue has been identified as a potential research problem. However, other researchers have looked into this problem from different perspectives. The different approaches that have been considered are concisely reported in the next paragraphs.

### 3.1. Why Is Energy Efficient Channel Coding Important for NB-IoT

One of the most important issues in the design of NB-IoT systems is error correction. If well designed, the channel coding technique for NB-IoT can help save a considerable amount of energy by significantly reducing the number of required retransmissions. This justifies the fact that several research studies have proposed channel coding techniques to achieve energy efficiency. In its current form, the NB-IoT uplink baseband processing can be divided into channel coding and modulation. In the case of the NB-IoT uplink, the channel coding includes Cyclic Redundancy Check (CRC) generation and attachment, turbo or convolutional coding, and rate matching. Reliability is a key performance criterion in any form of wireless communication including the NB-IoT. It is therefore crucial that any NB-IoT design ensures that end-to-end communication occurs reliably between terminals. As in most engineering designs, the effort to enhance one performance often comes with an associated cost in terms of the other. Channel coding is often used in most communication systems to ensure resilience to channel impairments and ensure reliable link transmissions. Therefore, previous research work has proposed various channel coding approaches to ensure reliability. The main identified approaches have been the traditional repetition approach and the Narrowband Link Adaptation (NBLA) approach [3]. Despite their attempt to enhance communication reliability, the approaches have been shown to suffer significant energy costs. This issue is what the present research work addresses. It strives to find a balance between ensuring reliable communication on NB-IoT uplinks while maintaining energy efficiency.

### 3.2. Existing NB-IoT Energy-Efficient Channel Coding (CC) Approaches

From the survey of the literature, the following main approaches provide a summary of the approaches that have been selected based on their relevance,

1. **Automatic Repeat Request (ARQ) Approaches**
  In this category of approaches, the receiver requests re-transmission of data packets if errors are detected, using some error detection mechanisms. Authors in [27] proposes an open-loop forward error correction technique for NB-IoT networks to optimize ARQ signaling. In this approach, signaling only needs to indicate the DL data transfer completion, and does not have to be specific on which particular Packet Data Units (PDU) are lost during the transmission. This allows for the reduction of the simplicity of the channel coding approach, and therefore, allows the saving on computational energy consumption. This approach has been demonstrated to be efficient in enhancing the data rate performance in the Downlink (DL) of the NB-IoT network. Due to its low complexity, this approach has been further proven to also enhance the energy efficiency of the NB-IoT network as it considerably reduces the computational energy consumption due to data reception on the transceiver of the NB-IoT node.
  An approach proposed in [28] considers using a hybrid automatic repeat request (HARQ) process in scenarios where the NB-IoT network can only support half-duplex operations. The HARQ approach has been demonstrated to be capable of reducing the processing time at the NB-IoT node. The obtained results in [28] demonstrate that the use of the HARQ approach can lead to a saving of up to 20% on the overall energy consumption of the network. This energy efficiency performance has also shown that it is not significantly affected by the increase in the scalability of the NB-IoT network when the HARQ approach is used.
  The authors in [29] propose a hybrid channel coding approach. It consists of signaling hybrid automatic repeat request (HARQ) acknowledgments for narrowband physical downlink shared channel (NPDSCH) and uses a repetition code for error correction. In this case, the User Equipment (UE) can be allocated with 12, 6, or 3 tones. However, the 6 and 3 tone formats are introduced for NB-IoT devices which due to coverage limitations cannot benefit from the higher device bandwidth allocation and results in higher energy consumption performance.
2. **Forward Error Correction (FEC) Approaches**
  Recent research ([30,31,32]) has investigated the efficiency of different re-transmission and FEC techniques in NB-IoT systems. Several researchers have quantified the effect of several network parameters on the efficiency of error correction techniques (and their associated network costs). However, no effort has yet been made to unify these studies into a systematic approach that could help with the selection of the most effective technique given certain network conditions.
  The authors in [33] have proposed an improved error correction algorithm for multicast over the LTE network and, by extension, over the Narrowband IoT network. The model used assumes a random distribution of packet losses and a constant loss rate in each scenario. The model can be expanded to include different error distributions and varying loss conditions during a series of NB-IoT downlink transmissions. The results obtained demonstrate that the use of a hybrid approach (combined HARQ and FEC) outperforms both the HARQ method as well as the FEC approach when used individually, in terms of energy efficiency.
  The authors in [34] proposed the use of an open-loop forward error correction technique as a mechanism to not only enhance the energy efficiency of the NB-IoT network but also to concurrently achieve efficient downlink data rate performance. The benefit of this approach lies in the fact that it enables extremely reliable firmware downloads which is an important feature in IoT in a number of applications such as wireless sensor network applications.
  Another Forward Error Correction channel coding approach for Narrowband IoT as proposed in [35] has been specifically designed to reduce the number of re-transmission attempts. This is mainly because it has been identified and demonstrated by [8,36,37] that most energy consumption in the Internet of Things and Wireless Sensor Networks (WSNs) is consumed through the transmission and the reception phases.
  Another unique Forward Error Correction approach which is based on algebraic-geometric theory compares the BER performance of algebraic-geometric codes and Reed-Solomon codes at different modulations schemes over additive white Gaussian noise [38]. In this approach, it is found that there is an improvement in terms of BER performance improvement at the cost of high system complexity when algebraic-geometric codes and the Chase-Pyndiah’s algorithm [39] are used in conjunction with each other.
  The authors in [40,41] propose a new enhanced Parallel Conventional Channel Coding (PCCC) approach which proposes the use of two or more convolutional encoders in parallel by means of turbo codes, demonstrates the ability to significantly reduce the overall number of received error packets as compared to the typical Serial Conventional Channel Coding (SCCC) as well as the typical PCCC. This channel coding approach can be of benefits in terms of enhancing the overall energy efficiency performance of the NB-IoT systems using the OFDM scheme, provided that its iterative decoding is computationally reliable and implementable on low resources processors of NB-IoT nodes with a restricted number of feedbacks.
  On the other hand, authors in [42] propose an enhanced OFDM platform based on Turbo Coding (TC) techniques. This approach considers the presence of a noisy channel (AWGN, Phase noise, Rician noise, ITU PA3) and demonstrates the ability to overcome its impairments to achieve excellent BER performance. Due to the high-reliability performance of the proposed approach as well as its use with the OFDM modulation scheme (used in LTE systems), it can be a suitable channel coding method for NB-IoT systems provided that its energy consumption performance also demonstrates to be efficient for sensor nodes NB-IoT type of applications [43].

Two main channel coding and uplink link adaptation schemes can be found in the literature. The first one termed an MCS-dominated approach, in which the MCS level is first adjusted based on feedback signals, after which the repetition number is adjusted. The second one is the repetition-dominated approach in which the focus is first on determining the appropriate repetition number based on the predefined NB-IoT network design criteria and then focusing on selecting the MCS level using the currently determined repetition number as part of the decision criteria. Apart from these two dominating approaches, there exist other approaches in literature such as the cooperative approaches [44] in which the impact of uplink interference on resources (energy, spectral efficiency, etc.) utilization efficiency is investigated each time before making transmission decisions by exploiting the cooperation among base stations which needs to be already designed.

### 3.3. Efficient Selection of Modulation Coding Scheme (MCS)

The design of an energy-efficient channel coding scheme for NB-IoT is directly linked to the selection of an appropriate modulation coding scheme (MCS). In order to achieve long-range communication, some work on efficient NB-IoT designs found in literature [3,21,45,46] have proposed efficient techniques for modulation scheme selection. The common idea behind most proposed approaches consists of trading off high data rates for higher energy in each transmitted bit (or symbol) at the physical layer (PHY). This design technique allows for a signal that is more immune and that can travel longer transmission distances. Therefore, in general, the identified aim of most designs is to achieve a link budget of 150±10 dB which can translate into a few kilometers and tens of kilometers in urban and rural areas respectively [3]. The reason why a value of 150 dB is used because it represents the lowest possible detectable radio signal power by the most sensitive possible radio receivers with noise sensitivity levels as low as −150 dBm which translates to 10-6 pico-watt reception power as per the following mathematical deduction,

(22) $$\left\{\begin{array}{c} -150=10\times log(\frac{P_{Rx}}{1\;mW})\\ \frac{P_{Rx}}{10^{-3}}=10^{-15}\\ P_{Rx}=10^{-18}\;Watts=10^{-6}\;picoW \end{array}\right.$$

These types of radio receivers are often used in the most sophisticated applications such as telemetry applications for example [47].

Encoding more energy into signal’s bits (or symbols) results in very high decoding reliability on the receiver side. Typical receiver sensitivities could, therefore, be as low as −130 dBm. Modulation techniques used for most LPWAN technologies can be classified into two main categories namely narrowband techniques and spread spectrum techniques. Spread spectrum techniques spread a narrowband signal over a wider frequency band but with the same power density. The actual transmission is a noise-like signal that is harder to detect by an eavesdropper, more resilient to interference, and robust to jamming attacks (secure) [48].

As opposed to other LPWAN technologies such as the LTE Cat-M1, which mainly uses spread spectrum modulation techniques, most work on NB-IoT designs found in literature [3,45,48], propose the use of narrowband modulation techniques. In general, narrowband modulation techniques provide a high link budget which is often less than 25 kHz. They are very efficient at frequency spectrum sharing between multiple links and they experience very low noise levels within each narrow band. To further reduce the experienced noise, some LPWAN technologies such as SIGFOX, WEIGHTLESS-N, and TELENSA, use ultra narrowband (UNB) of width as short as 100 Hz [45]. They are, therefore, susceptible to achieving longer transmission ranges.

One of the major differences between narrowband modulation techniques and spread spectrum techniques remains that spread spectrum techniques often require greater processing gain on the receiver side to decode the received signal(below the noise floor) while no processing gain is required to decode the signal at the receiver through frequency de-spreading for the case of narrowband modulation techniques. This results in simpler and less expensive transceiver designs. Different variants of spread spectrum techniques are used by existing standard LPWAN technologies such as Chirp Spread Spectrum (CSS) and Direct Sequence Spread Spectrum (DSSS).

### 3.4. Repetition-Dominated Channel Coding Approaches

In the repetition-dominated method, the repetition number is first adjusted based on the feedback ACK/NACKs, and then the MCS level is updated. Repetition is the key solution adopted by most NB-IoT designs to achieve enhanced coverage with low complexity. On the other hand, for one complete transmission, the repetition of the transmission is required to be applied to both data transmission and the associated control signaling transmission. Therefore, in most NB-IoT systems, before each NPUSCH transmission, the corresponding control signal data of which includes the Resource Unit (RU) number, the chosen MCS, and repetition numbers are required to be sent via the Narrowband Physical Downlink Control Channel (NPDCCH) [3]. The sequence of transmission with repetition during a single transmission is illustrated as given below.

**Figure 2:**
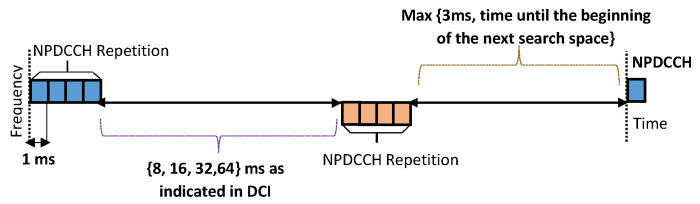
Illustration of data repetition during a single transmission.

Figure 2 illustrates the concept of repeated transmission in NB-IoT where both the NPDCCH and the NPUSCH transmission blocks with the same content highlighted using the same color are repeated four times in the duration of a single transmission. It is also important to highlight that according to the 3GPP TS 36.211 standard [49], the repetition number for the same block for NB-IoT can only be selected from 1, 2, 4, 8, 16, 32, 64 or 128.

The selection of a repetition number from the set of $2^{n}$ with *n* from 0 to 7, the process follows a normal network automatic repeat request (ARQ) as defined by the 3GPP rel. 13 in [5]. On the first successfully decoded uplink packet reception, the BS assesses the perceived radio quality in terms of received signal power. The received power parameter is also used as an input to the scheduler to decide which of the available 8 repetition numbers to use and it is reported back to the NB-IoT node which uses that repetition number till the next time, it experiences a failed transmission. At that moment, another repeat request packet is issued in order to define a new transmission repetition number. It can be noticed that the traditional repetition approach, the repetition number is not only selected from a limited set of numbers and also does not reduce should channel conditions improve. This is one of the drawbacks that the proposed EEACC approach has identified and attempts to mitigate.

### 3.5. The NBLA and Its Open-Loop Power Control Approaches

The NB-IoT link Adaptation (NBLA) approach is mainly focused on an inner loop link adaptation aspect that is mainly focused on addressing the issue of rapid changes that are often observed with the transmission BLER in NB-IoT systems. The NBLA approach as proposed in [3] works as follows.

- During the duration of a single period of *T*, all transmission ACK/NACKs are computed to work out an estimated value of the BLER,
- Based on the obtained BLER value, the transmission repetition number is adjusted accordingly to cope with the variation in the channel’s condition and to ensure that there is less probability of failed transmission.
- This is a way to save on energy consumption of the NB-IoT network.

The main challenge faced by the NBLA approach resides in the fact that despite its effort in estimating the channel condition based on the computed BLER value, because of the reduced (narrow) bandwidth and considerably unstable channel conditions of the NB-IoT systems, the NBLA power control strategy often fails to ensure reliable uplink transmissions. This results in significant energy wastage due to repeated unsuccessful transmissions. The development of a new and more energy efficient approach that is capable of adaptively addressing the variations of channel conditions by looking at more than one-dimensional aspect of previous BLER performance but also considering the MCS level in selecting an appropriate repetition number is highly needed. This is expected to contribute towards adequate energy management within NB-IoT systems.

### 3.6. NBLA Open-Loop Power Control

In the uplink scenario, the NB-IoT network normally only supports an open-link power control as stated in [49]. The reason for this open-loop power control exclusivity is mainly motivated by limited energy and processing capacity of most NB-IoT nodes such as sensor nodes which run on batteries most of the time. How this open loop power control is implemented within the NB-IoT nodes is that, based on the MCS and RU information alone. The NB-IoT node works out an estimate of the required power necessary to achieve an uplink transmission. This means that the Base Station (BS) (eNB in the case of LTE) does not send any form of power control command (information) to the NB-IoT before the uplink transmission.

According to the authors in [49,50], the transmit power ($P_{NPUSCH,c}\left(i\right)$) required by a NB-IoT node within a Narrowband Physical Uplink Shared channel (NPUSCH) during an uplink session within a given uplink slot *i* for serving a cell *c*, given that the number of repetitions of the allocated NPUSCH RUs is less than 2 can be modelled as

(23) $$\begin{array}{c} P_{NPUSCH,c}\left(i\right)=min\left\{P_{CMAX,c}\left(i\right),10\times log_{10}\times \left(M_{NPUSCH,c}\left(i\right)\right)+P_{O\_NPUSCH,c}\left(j\right)+\alpha_{c}\left(j\right)PL_{c}\right\} \end{array}$$

where

- $P_{CMAX,c}\left(i\right)$ is the configured NB-IoT node uplink transmit power in slot *i* for serving cell *c*,
- The possible values for $M_{NPUSCH,c}\left(i\right)$ are $\left\{\frac{1}{4},1,3,6\;\mathrm{or}\;12\right\}$ as defined in [50],
- $P_{O\_NPUSCH,c}\left(j\right)$ is a parameter composed of the sum of two components from the higher layers within the NPUSCH data re-transmission channel model,
- $PL_{c}$ is the downlink path loss estimate calculated in the UE for serving cell *c*, and
- $\alpha_{c}\left(j\right)$ is a coefficient configured by higher layers based on the estimated total loss over the link

Should the number of repetitions of the allocated NPUSCH RUs be higher or equal to 2, then, the $P_{NPUSCH,c}\left(i\right)$ can be simply modelled as

(24) $$P_{NPUSCH,c}\left(i\right)=P_{CMAX,c}\left(i\right)$$

In summary, it is important to note that none of the existing approaches (neither traditional repetition schemes nor the NBLA) consider the channel conditions in the selection of the repetition number for the traditional repetition schemes as well as dynamically minimizing this value based on the improvement of the channel conditions. At the same time, the existing NBLA scheme does not dynamically vary the Modulation Coding Scheme number based on the channel conditions. This is where the contribution of this study comes in.

## 4. The Proposed Adaptive Channel Coding Technique

The objective of the EEACC approach is to design an appropriate link adaptation scheme integrated with a proper selection mechanism of repetition number and MCS for the NB-IoT systems solely based on the aim of achieving greater energy efficiency, transmission distance, and high throughput while maintaining high transmission reliability.

The channel coding approach proposed by the present study is a 2-dimensional (2D) link adaptation approach which translates to a dual-objective optimization problem that aims at enhancing the NB-IoT network coverage without compromising on its energy efficiency performance. The proposed adaptive channel coding technique is twofold. It is composed of an inner loop and an outer loop adaptation scheme both aimed at enhancing the energy efficiency as well as the throughput of the network. In particular, the inner loop adaptation scheme is designed based on the channel conditions to guarantee transmission reliability and consequently, enhance the data rate of the network. On the other hand, the outer loop scheme is designed based on the Modulation Coding Scheme (MCS) number and the transmission repetition number.

Because the channel conditions of NB-IoT systems are known to be quite unstable [51] as their transmission block error ratio (BLER) rapidly changes, the present approach introduces an inner loop link adaptation procedure which focuses on dynamically varying the transmission repetition number based on a periodically sampled and estimated channel condition that is quantified based on the BLER performance. The current BLER performance is used to predict the channel conditions each time on the next transmission based on the Sequential Channel Estimation in the Presence of Random Phase Noise in NB-IoT Systems as proposed by [52]. In this channel estimation model, the main consideration is that although the coherent-time of the fading channel is assumed fairly long due to the assumed low mobility of NB-IoT user-equipments (UEs). Therefore, phase noises are considered before combining the channel estimates over repetition as a mechanism to improve the accuracy of the approach.

With phase noise $\phi_{l}\left[n\right]$ caused by oscillator fluctuations and a residual FO $f_{e}$ normalized by the sub-carrier frequency, the time-domain baseband received signal at the *n*th sampling time of the *l*th orthogonal-frequency-division-multiplexing (OFDM) symbol can be expressed as

(25) $$s_{l(Estimated)}\left[n\right]=e^{j\phi_{l}\left[n\right]}\left(\frac{1}{sqrtN}\sum_{k=-N/2}^{(N/2)-1}S_{l}\left[k\right]e^{j2\pi n(f_{e}+kN}\right)*h_{l}\left[n\right]+w\left(n\right)$$

where “∗” denotes the linear convolution, $S_{l}\left[k\right]$ is the transmit symbol on the *l*th OFDM symbol and the *k*th sub-carrier, h[n] is discrete fading channel taps, w(n) is additive-white Gaussian-noise (AWGN), and N is the Fast-Fourier-Transform (FFT) size.

The purpose of the inner loop link adaptation is to guarantee the transmission BLER to the target. Accordingly, the former is referred to as outer loop link adaptation that is inclusive of MCS level selection and repetition number determination. The proposed link-adaptation method is presented as follows.

### 4.1. The Inner Loop Approach

As discussed in a previous section, the inner loop link adaptation approach is designed to handle the rapid transmission of BLER fluctuations. This proposed inner loop approach works as described below:

- In a single period *T*, all transmissions of both positive and negative acknowledgments (ACK and NACKs) respectively are computed to work out the average BLER for the period which is then recorded for the specific period. Specifically, the appropriate evaluation period *T* for LTE systems is chosen to be in the order of tens of milliseconds while hundreds of milliseconds are used for the NB-IoT systems. This selection is mainly motivated by the realistic expected traffic rate on the LTE systems being normally higher than the NB-IoT counterpart.
- At the end of the considered period, the obtained BLER value is passed to the outer loop and used as a parameter in the selection of the appropriate transmission repetition number as clearly labeled in the description of the algorithm as presented in Algorithm 1,
- If the current BLER (the one of the present period) is found to be less than 7%, the repetition number for the next transmission should be decreased because it means that the channel conditions are good and therefore, the probability of successful transmission is high since there are fewer channel impairments.
- On the other hand, if the BLER is found to be between 7% and 13%, the channel is considered to be in a medium condition state. The proposed link adaptation approach considers that the repetition number should be maintained during the next period.
- Finally, if the current BLER is greater than 13%, the channel is considered to be in bad conditions, and therefore, the probability of successful transmission is reduced. This requires that the number of transmission repetitions must, therefore, be increased to guaranty a certain level of transmission reliability.

### 4.2. The Outer Loop Link Adaptation Approach

The outer loop link adaptation approach consists of the MCS level selection which is performed as described below:

- If a certain number of ACKs are successively successfully decoded at the NB-IoT receiver, then the MCS level is increased.
- On the other hand, when a certain number of NACKs are successively decoded at the NB-IoT receiver, then the MCS level is decreased.

In general, the number of ACKs is greater than that of NACKs to ensure a slow increase of the MCS level with ACK feedback and a quick decrease of the MCS level with NACK feedback. Due to the narrowband and low data rate for NB-IoT systems, the settings for LTE systems may not be applicable. Therefore, two aperiodic and event-based actions are defined for the EEACC approach namely the fast upgrade (FUG) and the emergency downgrade (EDG). In the event of FUG, the MCS is increased by one while in the event of an EDG it is decreased by one. Thus, the EEACC approach introduces a compensation factor ΔC(t) which can be modelled as

(26) $$\Delta C\left(t\right)=\left\{\begin{array}{c} min\;\{\Delta C\left(t-1\right)+C_{stepup},\Delta C_{max}\},\\ \;\;\;\;\;\;\;\;\;\;\;if\;HARQ\;\;feedback=ACK;\\ max\;\{\Delta C\left(t-1\right)-C_{stepdown},\Delta C_{min}\}\\ \;\;\;\;\;\;\;\;\;\;\;if\;HARQ\;\;feedback=NACK;\\ \Delta C(t-1),\\ \;\;\;\;\;\;\;\;\;\;\;if\;HARQ\;\;feedback=N/A; \end{array}\right.$$

where

- $\Delta C_{max}$ and $\Delta C_{min}$ are the upper and lower limits of the compensation factor ΔC(t),
- $C_{stepup}$ and $C_{stepdown}$ are the incremental compensation step sizes which is modelled as per the formula,
  (27) $$C_{stepdown}=C_{stepup}\times \frac{1-{BLER}_{target}}{{BLER}_{target}}$$
- N/A implies discontinuous transmission (DTX) which happens when the eNB does not detect any NPUSCH signal.

Various values for simulation parameters are selected within the simulation of the proposed algorithm. The choice of these parameter values is motivated each time by the objective of making the simulation process as realistic as possible. These parameter values include,

- A targeted Block Error Rate (BLER) value of 10%: The choice of this targeted BLER value is guided by the 3GPP standard Release 13 [49]. This targeted BLER value is considered to be the normal out-of-sync error rate condition for LTE/4G technology during Radio Link Monitoring (RLM) [53].
- The 7% and 13% threshold are typical values for specifying the channel state in an NB-IoT system to be good, medium, or bad. This choice is directly linked to the objective of maintaining the ±3% margin around the targeted 10% BLER as per the 3GPP standard specifications [49].
- The $C_{stepup}$ and $C_{stepdown}$ values are the incremental compensation step sizes. As in any iterative process, there is a need to choose a reasonable initial step size. The authors have chosen an initial Cstepup value and not a Cstepdown one since the $C_{stepdown}$ value is modeled first as dependent on being $C_{stepup}$.
- The initial value of 0.2 for the $C_{stepup}$ parameter is used to ensure that the MCS level is stepped down when it experiences an initial 20% probability of communication error. This is because 20% is the maximum 3GPP standard value for BLER when using LTE/4G Technology.

The proposed algorithm can be summarized as given in Algorithm 1.

The inner loop part is described between lines 2⟶8 of the pseudo code as presented by the pseudo-code in Algorithm 1. In this section, the Block Error rate (BLER) for each encoded block is checked every predefined *T* period time, against the threshold values of 7% and 13% for good, medium and bad channel states. Depending on the observed type channel condition (good, medium or bad), the channel repetition number *N* is either reduced to half, progressively increased by one or doubled respectively.

The pseudo-code for the outer loop section is described in lines 3⟶52. The outer loop consists of three scenarios namely,

- When the Modulation Coding Scheme (MCS) value is between the two predefined threshold values $\left(L_{min}<L<L_{max}\right)$. In this situation, the compensation value ΔC) is updated to the lowest or highest value based on whether the transmitter receives positive feedback (ACK) or negative feedback (NACK). The MCS level is increased by one or reduced by one accordingly.
- When the MCS reaches the minimum value ($L_{min}$), then based on the type of feedback received by the transmitter (ACK or NACK) and the position of current repetition number *N* in relation to the minimum predefined value $N_{min}$ and $N_{Max}$, a new MCS level is defined with an increase of 1 or maintained. The transmission repetition number *N* is reduced by half or doubled accordingly.
- Similarity, when the MCS value reaches the maximum value $L_{Max}$, then based on the type of feedback received by the transmitter (ACK or NACK) and the position of current repetition number *N* in relation to the minimum predefined value $N^{min}$, a new MCS level is with a decrease by 1 or maintained accordingly.

It is important to note that the validity of the proposed EEACC approach is based on the accuracy of our channel conditions assessment. Some key characteristics of this channel condition assessment have been empirically defined and standardized under the 3GPP standard release 13. The parameter ΔC(t) as used within the proposed approach plays the role of a channel characteristic compensation value. This approach is used to determine what level of channel noise and interference need to be suppressed from the current assessed channel conditions to move either to the classification as bad, medium, or good condition as defined in the standard. The $C_{stepdown}$ and $C_{stepup}$ parameters, therefore, represent the exact channel coding scheme reduction or increase needed to achieve the ΔC(t) channel compensation.

The choice of N/2 (half of the current repetition value) is a starting value for the algorithm to run from. This is done to represent a scenario in which the error rate has reduced (channel conditions have improved). The repetition number by half as a way to reduce re-transmission attempts and improve energy efficiency. If the reduction is found to be too large, the algorithm will self-adjust the value back to a higher value than N/2. Similarly, in the medium channel condition, the repetition number by 1 as a starting point. Naturally, the algorithm will swing between the repetition numbers in one of the two other cases. In the same way, when the channel condition is deemed to have worsened, the repetition number as a starting point is doubled as an extreme case if it is experienced to be too large, it will naturally self-readjust on the next transmissions.

**Algorithm 1:** The proposed EEACC pseudo-code1: **Initialization**: ${BLER}_{target}=10\%$, $C_{stepup}=0.2$, $C_{stepdown}=C_{stepup}\frac{1-{BLER}_{target}}{{BLER}_{target}}$, Δ C=0, $\Delta C_{max}=+5,\Delta C_{min}=-5$, MCS level *L* and its bounds $L^{max}$, $L^{min}$, repetition number *N* and its bounds $N^{max},N^{min}$. We empirically initialize the MCS level and repetition number based on the channel condition. 2: **if** period T expired out **then** 3:       **if**
BLER<7%
**then** 4:         N=N/2 5:       **else if**
7%<BLER>13%
**then** 6:         N=N+1 7:       **else if**
BLER>13%
**then** 8:         N=2N 9:       **end if** 10: **end if** 11: **if**
$L>L^{min}\&L<L^{max}$
**then** 12:      **if** HARQ feedback=ACK **then** 13.        $\Delta C=min\Delta C+C_{stepup},\Delta C_{max}$ 14.      **else if** HARQ feedback-NACK **then** 15.        $\Delta C=max\Delta C-C_{stepdown},\Delta C_{min}$ 16.      **end if** 17.      **if**
$\Delta C=\Delta C_{max}$
**then** 18.        L=L+1 19.      **if**
$\Delta C=\Delta C_{min}$
**then** 20.        L=L−1 21.      **end if** 22. **else if**
$L=L^{min}$
**then** 23.      **if** HARQ feedback=ACK **then** 24.        **if**
$N=N^{min}$
**then** 25.           $\Delta C=min\Delta C+C_{stepup},\Delta C_{max}$ 26.           **if**
$\Delta C=\Delta C_{max}$
**then** 27.              L=L+1 28.           **end if** 29.        **if**
$N>N^{min}$
**then** 30.           N=N/2 31.        **end if**
32.        **else if** HARQ feedback=NACK **then** 33.           **if**
$N=N^{max}$
**then** 34.              the current channel condition is very bad 35.           **else if**
$N<N^{max}$
**then** 36.              N=2N 37.           **end if** 38.        **end if** 39. **else if**
$L=L^{max}$ 40.      **if** HARQ feedback=ACK **then** 41.          **if**
$N=N^{min}$
**then** 42.             the current channel condition is very good. 43.          **else if**
$N>N^{min}$
**then** 44.             N=N/2 45.          **end if** 46.      **else if** HARQ feedback=NACK **then** 47.             $\Delta C=max\Delta C-C_{stepdown},\Delta C_{min}$ 48.             **if**
$\Delta C=\Delta C_{min}$
**then** 49.               L=L−1 50.             **end if** 51.      **end if** 52. **end if**

It is also quite critical to mention that, although small, the execution latency of the proposed algorithm has been assessed to form part of the overall up-link communication. This execution duration could be assumed to be quite negligible simply because of the remarkable progress that we now observed in the processing speed of nowadays micro-controllers. With the capability to be interfaced with clock frequencies of few dozens of MHz, nowadays microcontrollers that form the processor of the NB-IoT nodes, could have sampling speed of up to 70 MHz meaning sampling times of about 14.3 ns [54]. However, for our proposed research, the computational latency has been assessed as follows,

- Let us assume that the CPU of the NB-IoT node’s processor is running at a clock frequency of $f_{clk}$,
- Let also assume that the CPU has a machine cycle equal to *m* clock pulses (periods).
- The duration of a single machine cycle can be computed as $T_{mc}=\frac{m}{f_{clk}}$.
- Going from the principle that for a multi-cycle Microprocessor without Interlocked Pipelined Stages (MIPS) which considers the following five types of instructions with their respective number of machine cycles,
  1. Load (5 cycles)
  2. Store (4 cycles)
  3. R-type (4 cycles)
  4. Branch (3 cycles)
  5. Jump (3 cycles)
- By using a MATLAB R2019b Instruction Classification Learner application (Table 1), it was found that the program has the algorithm implementing the EEACC algorithm has an average total instruction count of n=15 instructions and the following percentages of instructions per type.
  This leads to an average of Cycles per instructions (CPI) of $CPI=\frac{5\times 52+23\times 4+14\times 4+9\times 3+2\times 3}{100}=4.41$ The execution time of the algorithm can be computed as follows:
  (28) $$T_{Ex}=CPI\times n\times T_{mc}=4.41\times 15\times \frac{m}{f_{clk}}$$
  This means that for the implementation on a typical NB-IoT node with a basic cheap microcontroller such as the PIC16F627A running a on a external crystal oscillator with a clock frequency $f_{clk}=16$ MHz with m=4 clock cycles per machine cycle, the execution time of the EEACC algorithm can be computed as follows,
  (29) $$T_{Ex}=4.41\times 15\times \frac{4}{16\times 10^{6}}=16.5375\;\mu s$$
  This processing duration significantly reduces with faster modern microcontrollers. Depending on the type of applications for which the NB-IoT system is deployed as well as the cost constraints, ultra low power and faster microcontrollers such as the Silicon Laboratories EFM32 microcontrollers can be used to implement the EEACC algorithm along with full LTE protocol stacks for complex applications such as the control of unmanned aerial vehicles (UAVs) [55].

## 5. Performance Evaluation

### 5.1. Evaluation Setup

In order to assess and validate the performance of the proposed EEACC approach, various simulations were carried out under the NB-IoT network conditions summarized within the set-up parameters given in Table 2.

Furthermore, it is important to note that that a 4:1 Multiple Input Multiple Output (MIMO) with Alamouti decoding technique as well as an eNB antenna design is considered in these simulations.

As the study was carried out in the context of a Narrowband Internet of Things (NB-IoT) network deployed in an LTE cellular network, the Quadrature Phase Shift Keying (QPSK) LTE modulation scheme is considered. The eNodeBs in an LTE network is usually built to support QPSK, 16 QAM, and 64QAM modulation techniques for the Down Link direction. But considering that the end nodes (NB-IoT motes) are often computationally limited devices that are micro-controller based in many cases (sensor nodes, smart water meters, etc.), the choice of the modulation scheme is often oriented towards QPSK [56].

### 5.2. The EEACC MATLAB Simulation Approach

The proposed EEACC MATLAB simulation methodology consists of the following steps:

- First, a random NB-IoT network of nodes is generated on a planar using the uniform distribution functions of MATLAB.
- Then, the Base Station (BS) is positioned using the geometry middle point as root to generate a tree topology by the Dijkstra’s algorithm.
- A meshnet topology is formed as NB-IoT nodes are progressively deployed. Upon deployed, every node broadcasts a small interest message to identify its neighbours and path to the BS as guided by the Friis transmission equation. It is also important to mention that the Meshnet is the most preferred deployment approach for the NB-IoT networks [57,58]. This is due to the self-forming and self-healing nature of Mesh Networks which presents the following advantages when used for NB-IoT systems:
  1. Better efficiency: With its ability to quickly create robust ad-hoc networks, mesh technology is proven to be a high-performance data transfer solution which happens to be the core of most NB-IoT applications (example of Wireless Sensor smart metering NB-IoT networks and many more).
  2. Easier maintainability: Built-in diagnostics and fault reporting ensure you can respond to maintenance issues quickly and ensure continuity of service.
  3. Easy installation: The network builds as the NB-IoT system is deployed. This is very important for Internet of Things networks in general because one of the major challenges of IoT network deployment is its financial remuneration aspect as it takes millions of devices to be deployed for a network to start to become profitable. It is also important to mention that, the deployment of IoT networks follows a more contagious approach as more nodes connect to the network because of the demonstrated benefits of existing nodes. Therefore, the deployment of NB-IoT networks must follow a meshnet approach so that on a new installation of a new node, it can self-configure by identifying first its neighbouring nodes for relay purposes and secondly its associated Base Station.

**Figure 3:**
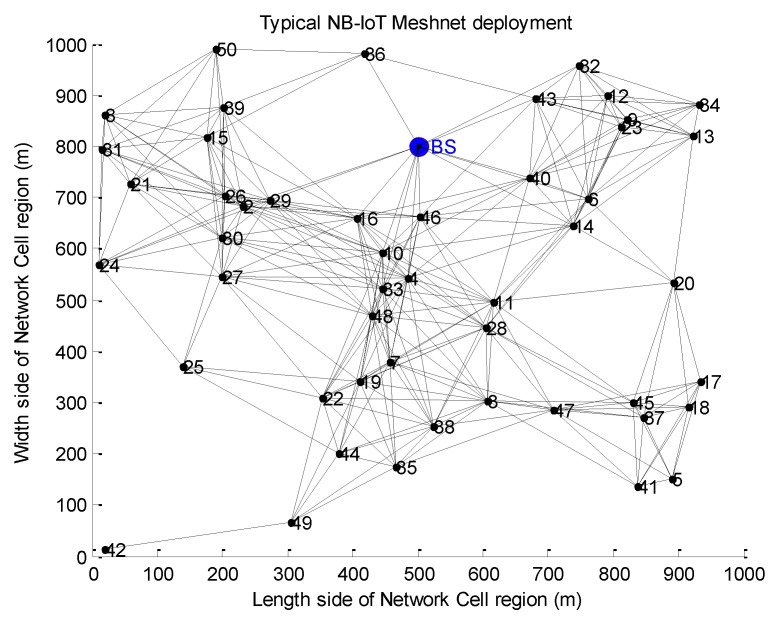
A typical Meshnet NB-IoT cell deployment.

A typical meshnet planar deployment scenario is represented in Figure 3.

- All the simulation parameters as described in Table 2 are defined.
- Each NB-IoT node is assumed to be running from a 4.5 V battery pack of 3 Lithium-Ion battery cells each of 1.5 V.
- Each NB-IoT node is assumed initially (upon network deployment) and for most of the time, to run its microcontroller chip in the sleep mode with only its time clock and NB-IoT radio interface external interrupt active to allow for radioactivity detection for possible data reception handling.
- A MATLAB timer class with an instantiated object for each NB-IoT node, based on the simulation computer’s clock is used to keep track of the timing function at each node.
- Based on a random uniform process, each NB-IoT node is due to send a single data packet at a predefined rate (could be hourly, daily, etc depending on the application at hand). For every single transmission, every single NB-IoT node described as an object of a defined class (mote), implements the EEACC algorithm as presented in Algorithm 1 for transmission of data packets.
- During the running of the simulation, every node maintains a history of its parameter values in the form of a table among which its residual energy value.
- At the end of the predefined simulation time, all the data from the different nodes are computed and compared for each of the three algorithms (traditional repetition algorithm, the NBLA as well as the proposed EEACC).

### 5.3. Obtained Results and Discussion

This section critically discusses and interprets the obtained results in light of the research objectives and goals of the research. It is important to note that the sequential channel estimation considered in the study as proposed in [52], takes into account the presence of random phase noises being present in the signal. The AWGN channel models the random phase noises in this study. The channel model used in the simulations considers a slow fading channel model where the coherence time of the channel is large relative to the delay requirement of the application. This is because real-time NB-IoT applications were not considered in this study which would require extremely small latencies. Although latency performance is part of what the study assesses, it is not the main design consideration. On the other hand, it is assumed that the nodes are not mobile which makes the proposed model time-invariant. An urban deployment set-up is considered where multi-path fading is assumed. The AWGN is simply modeling the random phase noise which forms part of the slow fading channel model that is used for the simulations of this study. For a fair evaluation of performance and comparison between the three considered schemes namely the NBLA, the traditional repetition approach as well as the proposed EEACC approach, all three systems make use of the same block size. As per the 3GPP TS36.213 standard [49], an NB-IoT device can select a downlink transport block size (TBS) on the MAC layer from a size of 2 bytes (16 bits) up to a size of 85 bytes (680 bits). In the case of the simulation, in this study an average block size of 44 bytes is used.

First, the reliability performance of the proposed approach is assessed in comparison to the traditional repetition approach as well as the NBLA approach. This assessment is performed by varying the transmission power of the different NB-IoT nodes and for the same number of transmissions, the average BLER value is computed for each of the three approaches.

**Figure 4:**
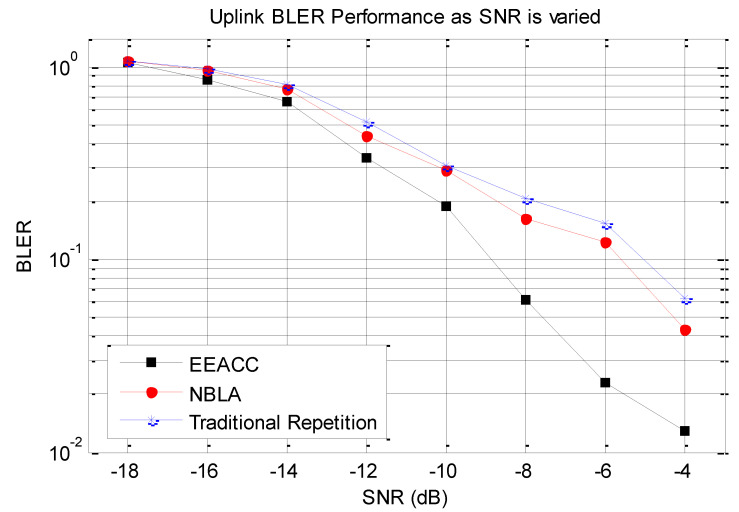
Uplink BLER Performance Comparison as Power is varied.

The obtained results as illustrated in Figure 4 clearly demonstrate that the Block Error rate (BLER) of the traditional repetition approach, the NBLA approach as well as the one of our proposed EEACC approach are all seen to reduce as the Signal-to-Noise Ratio (SNR) increases. This is an expected behaviour which materializes and confirms the emitted hypothesis that the higher the transmission power, the higher the amount of energy as encoded in a bit through modulation and channel coding, the higher the resilience to noise and other channel impairments. However, our focus is on observing the performance of the proposed approach in comparison to the other two approaches in terms of the extra contribution in terms of network reliability. In terms of further analysis, it can be observed that the slope of the EEACC decreases much faster than that of the others after an SNR of −14 dB. This is justified by the contribution of the repeated transmission capability of the EEACC coupled with its channel-aware MCS selection which demonstrates fewer transmission failures and therefore, further enhances the overall reliability performance of the network. These previous two justification factors are further emphasized by the increase in transmission power $P_{Tx}$ which enhances the SNR within a single transmission period *T* where the channel conditions remain more or less the same. The increase in the transmission power for the same noise floor level translates in a higher Signal-to-Noise Ratio (SNR). This is because the increase in SNR is directly proportional to the increase in the transmission power in terms of the SNR ratio value definition which translates in additional dB offset value. It is also important to observe that while for SNR values that range from −18 dB to −14 dB, all the BLER values are quite the same for the three approaches. Overall, the BLER values of the NBLA as well as the traditional repetition approaches are higher compared to the proposed EEACC approach for the various SNR values. This means that the EEACC exhibits better network reliability performance as compared to the NBLA and the traditional repetition approaches. Finally, it can be noticed that the BLER performance of the NBLA as well as the one of the traditional repetition approach, remains closer as transmission power is increased. Considering the EEACC approach, the BLER performance significantly drops faster as low as 0.18% for an SNR of −4 dB.

**Figure 5:**
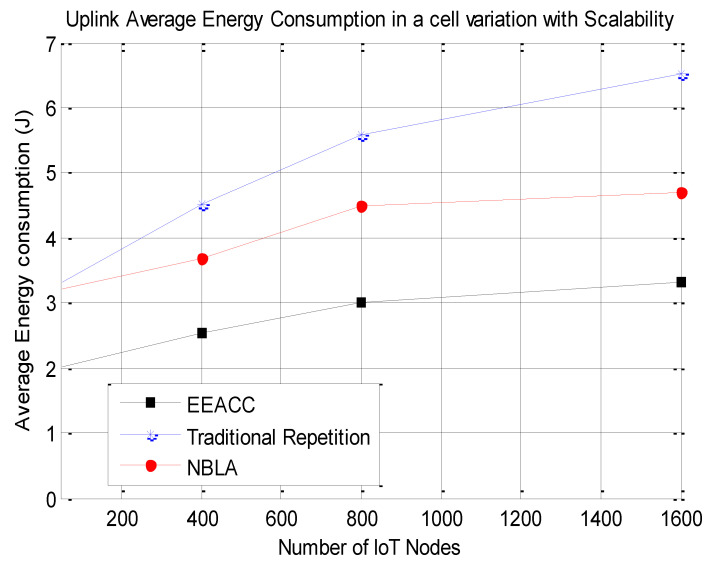
Uplink Average Energy consumption variation with increased scalability.

Secondly, the average energy consumption of the NB-IoT network was also computed as the number of NB-IoT nodes is varied with each of the three approaches, namely the traditional repetition, the NBLA as well as the proposed EEACC approach. The obtained results are depicted in Figure 5. Various simulations were conducted with various NB-IoT nodes as a mechanism to assess the impact of the network’s scalability on its energy efficiency performance for the different approaches. This is very important as it assesses the compatibility of the proposed EEACC approach with the nowadays massive IoT deployments in terms of maintaining an acceptable level of network performance.

It can be observed from Figure 5 that the EEACC approach is more energy-efficient than the NBLA and the traditional repetition approaches. In addition, the average energy consumption of all three approaches increases as the number of NB-IoT nodes is increased in the network. For example, given a total of 800 NB-IoT nodes considered within a cell, the proposed EEACC approach consumes 44.01% less energy compared to the NBLA approach and a 49.51% even less energy compared to the traditional repetition approach.

**Figure 6:**
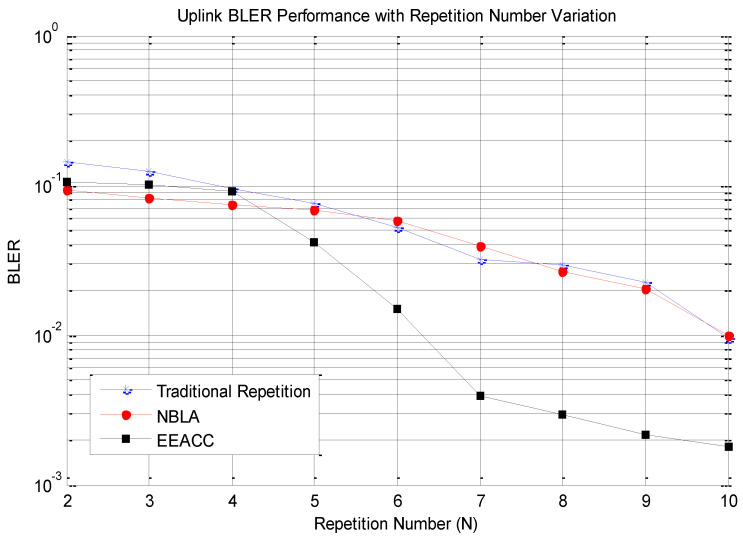
Uplink BLER Performance Behaviour with increased transmission repetition number.

Additionally, the behavior of the BLER performance is considered as the number of repeated transmissions was increased. This performance evaluation was aimed at assessing the impact of increasing the transmission repetition on the transmission reliability of the NB-IoT network. Therefore, the transmission power was kept constant at 0.1 W which translates in a constant SNR=−10 dB assuming that the channel conditions did not change significantly during a particular simulation period. The obtained results are given in Figure 6.

As seen in Figure 6, the BLER is almost half of what it was for an SNR of −10 dB as depicted in Figure 4 as the transmission repetition number is doubled (N=2). This translates to the fact that for doubling the number of transmission repetitions, the probability of failed block transmission is almost reduced by half. It can also be noticed that as the number of transmission repetitions increases. It is also observed that the BLER is reduced for all three approaches. It is important to notice that the BLER of the traditional repetition approach as well as the one of the NBLA approach remains close to each other as the number of transmission repetitions is increased. On the other hand, it is observed that in the case of the EEACC approach, it significantly reduces to as low as 0.18% as the repetition number *N* reaches the preset maximum value $N_{Max}=10$.

Finally, the average transmission delay on a link basis was also evaluated as the number of NB-IoT nodes was scaled up within a cell. This is to assess the impact of network scalability on the latency performance of the NB-IoT network for the proposed EEACC approach as mechanism to evaluate the applicability of the proposed EEACC method in real-time IoT applications where low latency performance is critical. The obtained results are shown in the following figure.

**Figure 7:**
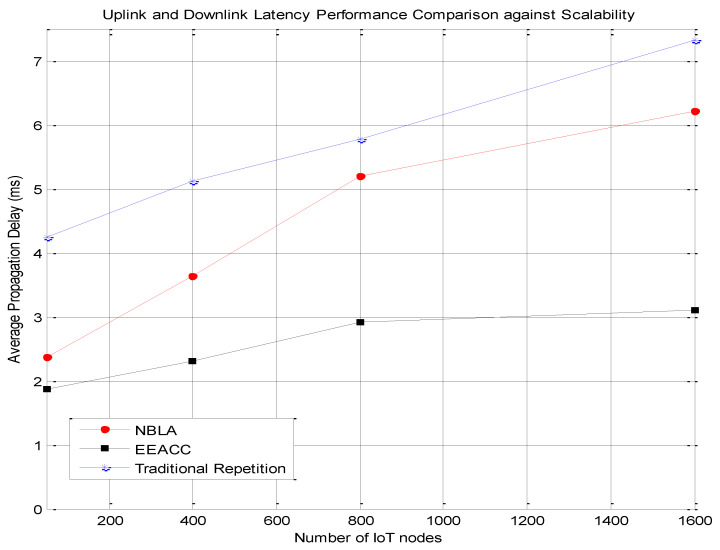
Uplink and Downlink Average full transmission delay with increased scalability.

As it can be noticed in Figure 7, despite its increased computational activities, when compared to the NBLA and the traditional repetition approaches, the EEACC still achieves lower latency on a link basis. This is mainly due to the advancement of nowadays processors, computational energy but also time is not significant as compared to the propagation delay in a wireless network of object nodes such as sensor nodes, etc. Once again, as it can be noticed the latency performance of the NBLA and the traditional repetition approaches are quite close despite the fact the NBLA still performs with less latency as compared to the traditional repetition approach.

## 6. Conclusions

A new proposed 2-D adaptive channel coding and link adaptation technique is presented in this manuscript which is relevant to reducing the energy consumption of NB-IoT systems in lights with the current massive, time-critical, and industry ultra-reliable requirements of IoT applications. Part of this work has been accomplished through extended bibliography search and reviewing on the NB-IoT channel coding and link adaptation schemes with, each time, a brief analysis of the advantages and disadvantages of each. The proposed EEACC approach is then step-by-step developed and presented. The obtained results and their discussion clearly highlights the major contributions of the proposed approach as they clearly demonstrate a much more resilience of the proposed approach to channel impairments, resulting in enhancing the network reliability performance. The proposed novel 2-D adaptive channel coding and link adaptation technique proves to minimize the overall energy consumption of the NB-IoT system and demonstrates the ability to sustain network scalability without significant increases in network latency. The observed performance of the proposed EEACC in terms of network scalability, latency, energy efficiency, and reliability metrics, in comparison with the existing traditional repetition approach as well as the NBLA approach is the key contribution of this research work. Future work plans to implement and assess the performance of the proposed EEACC algorithm on an NB-IoT embedded test-bed platform for a smart water consumption monitoring application.

## 7. Future Work

The proposed energy efficiency approach (EEACC) has been demonstrated to be an energy efficiency approach as compared to the traditional repetition and the NBLA approaches. Therefore, our proposed future research work consists of firstly implementing and assessing the proposed energy-efficient approach on-chip by using an application scenario such as smart water metering or environmental monitoring sensor network to further practically validate the proposed approach. Secondly, we propose to theoretically assess the solution of the problem using an optimization approach. 

## Figures and Tables

**Table 1:** MATLAB instructions classification application results.

Instruction Type	Percentage
Load	52%
Store	23%
R-type	14%
Branch	9%
Jump	2%

**Table 2:** Key Simulation parameters.

System bandwidth	200 kHz
Carrier frequency	900 MHz
Subcarrier spacing	15 kHz
Chanel estimation for NPDCCH	Sequential Channel Estimation in the Presence of Random Phase Noise [52]
Interference Rejection Combiner	MRC
Number of Tx antennas	1
Number of receive antennas	2
Frequency offset	200 Hz
$L_{min}$	4
$L_{max}$	12
$N_{min}$	2
$N_{max}$	10
Time offset period	2.5 μs
Network deployment Model	Mesh Network (Meshnet)
Channel Model	Time-invariant slow fading with random phase noise following an Additive White Gaussian Noise (AWGN) like distribution
slow or almost no nodes mobility considered	Static nodes No fading channel consideration.
LTE Modulation Scheme	Quadrature Phase Shift Keying (QPSK)

## Abbreviations

The following key abbreviations are used in this manuscript:

NB-IoT	Narrowband Internet of Things
NB-IoT node	Device equipped with a processor and an NB-IoT modem such as a sensor node, a smart water meter, etc.
NBLA	Narrowband Internet Link Adaptation
EEACC	Energy-Efficient Adaptive Channel Coding
LTE	Long Term Evolution
3GPP	Third Generation Partnership Project
SNR	Signal to Noise Ratio
NPUSCH	Narrowband Physical Uplink Shared Channel
BS	Base Station
RU	Resource Unit
MCS	Modulation Coding Scheme
BLER	Block Error Ratio
LPWAN	Low Power Wide Area Networks
ARQ	Automatic Repeat Request
FEC	Forward Error Correction
TBS	Transport Block Size
